# Humor and Quality of Life in Adults With Chronic Diseases: A Systematic Review

**DOI:** 10.7759/cureus.55201

**Published:** 2024-02-29

**Authors:** Eleni Bartzou, Evangelia Tsiloni, Stefanos Mantzoukas, Elena Dragioti, Mary Gouva

**Affiliations:** 1 Research Laboratory Psychology of Patients, Families & Health Professionals, University of Ioannina, Ioannina, GRC; 2 Research Laboratory Psychology of Patients, Families & Health Professionals,, University of Ioannina, Ioannina, GRC

**Keywords:** adults, chronic diseases, wellbeing, quality of life (qol), humor

## Abstract

Individuals grappling with chronic ailments often undergo a deterioration in their overall quality of life (QoL), encompassing psychological, social, and physical dimensions of well-being. Acknowledging that humor has demonstrated the potential to engender favorable effects on QoL, this systematic review endeavors to investigate the correlation between humor and QoL among adults contending with chronic health conditions. A comprehensive review of quantitative data was conducted in accordance with the Preferred Reporting Items for Systematic Reviews and Meta-Analyses (PRISMA) 2020 guidelines. PubMed/MEDLINE, PsycINFO, and Cumulative Index to Nursing & Allied Health (CINAHL) were comprehensively searched from the establishment of each database up to June 22, 2023. Furthermore, reference lists of the included datasets and pertinent review articles were scrutinized exhaustively. The Newcastle-Ottawa Scale (NOS) was employed to assess the quality of eligible studies. A total of 18 studies satisfied the inclusion criteria. These studies encompassed a diverse spectrum of chronic disease categories (including cardiovascular diseases, various types of cancer, etc.) and collectively involved a participant cohort comprising 4,325 individuals. Remarkable findings surfaced, indicating a noteworthy association between distinct facets of humor-such as one's sense of humor, coping humor, humor styles, and laughter-and psychological QoL. Nonetheless, the relationship between humor and physical QoL exhibited a more intricate pattern, characterized by mixed outcomes. Despite the limited and inconsistent evidence across studies, humor appears to exhibit a positive association with QoL.

## Introduction and background

Chronic diseases, also called non-communicable diseases (NCDs), are persistent and long-term conditions that usually progress slowly and are typically caused by genetic, physiological, environmental, and behavioral factors [1]. Chronic physical diseases such as diabetes, heart disease, cancer, and respiratory disease are the leading causes of death and morbidity worldwide [2]. According to the World Health Organization [3], over 41 million people die annually from a chronic disease, with cardiovascular diseases (e.g., stroke, heart attack) claiming the top spot by causing 17.9 million deaths each year. Chronic illnesses, therefore, represent a substantial public health challenge, exerting a continuous disruptive influence on the health and lifestyle of affected individuals [3]. The enduring nature of these conditions necessitates persistent management and treatment strategies, which may have far-reaching implications on multiple facets of daily life, encompassing physical functionality, emotional well-being, and the quality of social interactions [3].

The quality of life (QoL) of adult patients with chronic diseases has been investigated by numerous studies, contributing to a better understanding of the multifaceted effects of these health conditions [4,5]. QoL is a multidimensional concept encompassing an individual's overall well-being and satisfaction with various aspects of life, including physical health, mental and emotional well-being, social relationships, and environmental factors [6]. The term “Health Related QoL” (HRQoL), the self-perceived health status, is often used interchangeably with QoL, and it consists of three broad domains: physical, psychological, and social functioning [7]. Research has shown that living with a chronic illness, including cardiovascular disease [8,9], various types of cancer [10-14], stroke [15,16], and diabetes [17-19] can be significantly challenging for many aspects of QoL [20].

An important buffer against these physical, psychological, and social hazards can be humor [21]. Humor can be defined as a cognitive and emotional process that elicits amusement and offers a feeling of enjoyment and pleasure [22]. It is a complex, multifaceted phenomenon often characterized by the perception of something as amusing or funny, leading to expressions such as laughter or smiles [22]. Additionally, humor can be used as a coping strategy or defense mechanism to support individuals dealing with stressful or challenging situations such as chronic diseases [22]. More specifically, humor as a coping scheme serves as a vital psychological resource, offering individuals with chronic health problems a means of coping with their challenges with resilience and optimism [23]. Humor particularly acts as a stress reliever and mood enhancer [22], strengthens social bonds [23], and is associated with better immune function and pain tolerance through laughter [24]. Various instruments have been developed for assessing different aspects of humor, such as the Situational Humor Response Questionnaire - SHRQ [25], the Coping Humor Scale-CHS [26], and Brief COPE [27]. Studies have shown that humor enhances physical, mental, and social well-being, thereby promoting the overall quality of life [28-31]. Many health settings provide humorous material, and humor-based therapeutic programs to help patients reduce stress and boost their sense of well-being [32].

To our knowledge, no review has investigated the relationship between humor and quality of life in adults living with chronic diseases. Therefore, our systematic review aimed to provide a comprehensive evaluation of existing evidence regarding the association between distinct facets of humor (e.g., sense of humor, humor coping) and different domains of quality of life (e.g., physical well-being, life satisfaction, overall quality of life).

## Review

Methods

This systematic review followed the updated Preferred Reporting Items for Systematic Reviews and Meta-Analyses (PRISMA, 2020) guidelines [33]. The protocol is available online at: https://osf.io/gt6zr.

Search Strategy 

A systematic review approach was employed to identify studies exploring the relationship between humor and QoL in individuals dealing with chronic illnesses. Authors conducted an exhaustive search across three electronic databases, namely PubMed/MEDLINE, PsycINFO, and Cumulative Index to Nursing & Allied Health (CINAHL), without imposing any restrictions related to language, age, or setting. This search spanned from the establishment of these databases up to June 22, 2023.

The search algorithm utilized a combination of specific search terms, including "older adults," "humor," and "quality of life". To comprehensively identify relevant studies, authors employed Medical Subject Headings (MeSH), synonyms, and the Boolean operators "AND" and "OR." The full search strategy for PubMed/MEDLINE was "(elderly OR elder* OR older people OR older OR older person OR older adults OR gerontology) AND (humor* OR humour OR smile OR smiling OR happy OR laughter) AND (quality of life OR wellbeing OR well-being OR welfare OR wellness OR HRQOL OR health-related quality of life). Furthermore, the algorithm was tailored for each database to optimize the retrieval of pertinent studies.

Two independent reviewers (EB and ET) initially screened the articles based on their titles and abstracts. Full-text examination was performed for records that met the initial screening criteria. Notably, no studies exclusively focused on older adults while meeting the remaining inclusion criteria were found. Consequently, authors expanded the age group to encompass adults aged 18 years and above. Additionally, authors extensively reviewed the reference lists of the included full-text articles and explored relevant grey literature. In cases of discrepancies or disagreements, a third independent reviewer (MG) was consulted, and consensus was reached through discussion.

Eligibility Criteria

After the adjustment in age group, studies were considered eligible for inclusion if they: 1) involved adults (>18 years) with chronic physical diseases (e.g., diabetes, cancer, etc.); 2) evaluated the humor of participants (e.g., sense of humor, humor as a coping strategy); 3) assessed QOL/HRQOL and its dimensions (e.g., life satisfaction, psychological well-being, physical QoL) using a standard tool (e.g., Short-form 36 Health Status Questionnaire - SF-36 [34]), and 4) reported quantitative data on the association between humor and quality of life. Authors excluded studies that presented humor interventions or laughter therapy programs to provide a clearer and more focused analysis of the relationship between humor and QoL based on observational data, where humor acts as an exposure variable.

Data Extraction and Synthesis

Citations were gathered and imported into EndNote X9 software for organization. Duplicate entries were systematically removed, and the resulting list was then exported to an Excel spreadsheet. Subsequently, the evaluation of studies against inclusion and exclusion criteria was conducted by one of the authors (EB). The same author created an Excel spreadsheet with following information for the included studies: DOI, first author, year of publication, country, study design, sampling method, sample size, participant characteristics (mean age and sex), humor measure, humor component, QoL measure, QoL component, and results. The final data of eligible studies were reviewed and verified by four reviewers (ET, SM, ED, and MG). Given the methodological diversity across the included studies, it was not feasible to perform a quantitative synthesis, commonly referred to as a meta-analysis [35]. Hence, a non-statistical method (i.e., a narrative approach) was adopted to analyze and interpret the findings.

Risk of Bias (Quality) Assessment

The methodological quality of the included studies was evaluated using the Newcastle-Ottawa scale (NOS) [36]. NOS uses a star scoring system based on the components of subject selection, comparability, and assessment of outcome. An adaptation of the NOS was used to evaluate cross-sectional studies [37]. Regarding the comparability of each study, one star was given for studies controlled for covariates, such as gender, socioeconomic status, and other factors related to humor and QoL (e.g., lifestyle factors, psychological factors, health condition, etc.). Studies were granted a maximum of four points for selection (five points for cross-sectional studies), two points for comparability, and three points for exposure or outcome. A score of seven or higher indicated high quality. Two reviewers (EB and MG) independently appraised the risk of bias in eligible studies and any disagreement was resolved through discussion with a third reviewer (ED).

Results

Search Results

**Figure 1:**
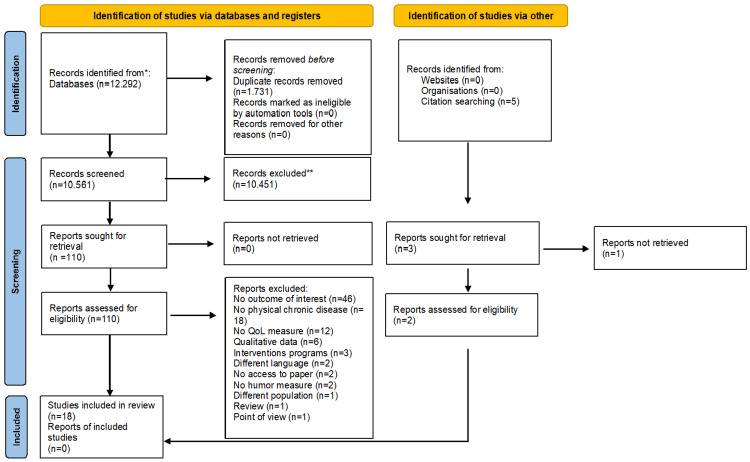
Preferred Reporting Items for Systematic Reviews and Meta-Analyses (PRISMA) flowchart Notes: n= number of studies, QoL=Quality of life

The electronic database searches resulted in 12,292 references; the elimination of duplicates (1731) led to 10,561 results. Five publications were identified using the hand search method. After title and abstract screening, 110 studies identified from databases and two studies identified from citation searching, were retained for full-text review. The full texts were then carefully read and assessed based on the inclusion and exclusion criteria. Finally, 18 studies (16 from electronic databases and two from citation searching) were found to be eligible. Figure 1 depicts the selection process in a PRISMA flowchart [33].

Characteristics of the Included Studies

The main characteristics and results of the 18 eligible studies were summarized in Table 1. The included studies were published between 2005 and 2019. Six studies were conducted in the USA (33%) [38-43], five studies in Norway (28%) [31,44-47], and one study each in Australia [48], Greece [49], Japan [30], Nigeria [50], Portugal [51], Sweden [52], and Switzerland [53]. The sample size of the included studies ranged from 22 to 1800 participants; eight studies had a sample size of less than 100, nine studies between 100 and 600, and the remaining one study had a sample size over 1,000. The chosen studies included a total of 4,325 participants. 

**Table 1:** Characteristics of studies Notes: CR=Cross sectional study, CO=Cohort study, n=number of participants, m=mean, NR=not reported, QoL=Quality of life, HRQoL=Health-related QoL, H&N QoL=Head and neck cancer quality of life, RCC= Renal Cell Carcinoma, CAD=Coronary artery disease, FS=Fibromyalgia syndrome, SS=Primary Sjögren’s syndrome, COPD=Chronic obstructive pulmonary disease, CKD=Chronic Kidney Disease, CRC=Colorectal cancer, HC=Humor coping, SHQ-SHQ6=Svebak Humor Questionnaire, SHQ-L=L Scale of SHQ, SHQ-M=M scale of SHQ, HSQ=Humor Style Questionnaire, SHRQ=Situational Humor Response Questionnaire, DSQ=Defense Style Questionnaire, CHS= Coping Humor Scale, UHI= Use of Humor Index, HO= Humor Orientation Scale, MSHS= Multidimensional Sense of Humor Scale, CCRC= Coping with Colorectal Cancer, EORTC QLQ-C30= European Organisation for Research and Treatment of Cancer Quality of Life Questionnaire,  EORTC QLQ-H&N35= European Organisation for Research and Treatment of Cancer Quality of Life Questionnaire Head and Neck Module, SF-36=The Short Form 36 Item Health Survey, PGWB= Psychological General Well-Being Index, WHOQOL-BREF=World Health Organization Quality-of-Life Scale – Brief, HAQ=Health Assessment Questionnaire, SWLG=Satisfaction with life in general, EQ-5D=EuroQol-5 Dimension Questionnaire, HRQOLISP=Health-related quality of life, FACT-C= Functional Assessment of Cancer Therapy, SWBI=Sense of Well-Being Inventory, SLS=Satisfaction with Life Scale, SF-12=The Short Form 12 Item Health Survey

Authors, Year	Country	Study design	Characteristics of sample	Chronic disease	Humor	QoL	Main outcomes	Secondary outcomes	Quality
Aarstad et al. (2011) [46]	Norway	CS	139 (m=60) Age range: <80 Males: 104, Females: 35	Head and neck cancer	Cope Questionnaire, SHQ - L	EORTC QLQ-C30, EORTC QLQ-H&N35	(1) Cope humor was significantly correlated with EORTC QLQ-H&N35 (r=.18, p<0.05), but not with EORTC QLQ -C30 function scale (r=-.08) and EORTC QLQ-C30 symptom scale (r=.06). (2) Sense of humor (SHQ-L) was correlated with QoL: EORTC QLQ:-C30 function scale (r=-.20, p<0.05), EORTC QLQ-C30 symptom scale (r=.21, p<0.05), and EORTC QLQ-H&N35 (r=.30, p<0.001).	Distress was significantly associated with sense of humor (r=.28, p<0.001), but not with coping humor (r=-01).	Good
Aarstad et al. (2008) [45]	Norway	CO	55 (m=56) Age range: <80 Males: 46, Females: 9	Head and neck cancer	COPE questionnaire	EORTC QLQ-C30, EORTC QLQ-H&N35	Coping by humor levels were correlated with the QoL sum score: QLQ-C30 global health/ QOL score (r=-0.34; p<0.05), QLQ-C30 functional sum score (r=-0.34; p<0.05), QLQ-C30 symptom scale score (r=0.29; p<0.05), EORTC QLQ-H&N35 cluster sum score (r=0.38; p< 0.01).	NR	Good
Aarstad et al. (2005) [44]	Norway	CO	Cancer:79 (Males) (m=59.9), Control: 63 (55.3) Age range: <80	Head and neck cancer	SHQ-L, SHQ-M	EORTC QLQ-C30, EORTC QLQ-H&N35	(1) The total humor score (SHQ-L and SHQ-M) measured at diagnosis predicted the global QOL level at follow-up (r= - 0.44, p <0.05). (2) With adjustment for neuroticism, the humor SHQ predicted the global QOL level (r=0.46, p<0.05), and the H&N QOL sum score (r= 0.45, p<0.05).	The total humor score measured at diagnosis predicted the “psycho” depression score at follow-up (r = 0.42, p<0.05) and total depression (r=0.37, p<0.1).	Good
Beisland et al. (2013) [47]	Norway	CS	185 (m=65.4) Age range: 34-84 Males: 127, Females: 58	Renal Cell Carcinoma (RCC)	COPE Inventory	EORTC QLQ-C30	Coping by humor was negatively associated with Functional HRQoL score (r=-0.18, p< 0.05), and positively associated with Symptom HRQoL (r=0.20, p<0.01).	NR	Good
Forrette (2019) [38]	USA	CS	69 (m=38.51) Males: 40 Females: 29	Coronary artery disease (CAD)	HSQ	SF-36	Humor styles did not significantly contribute predictive ability concerning either physical or mental QoL.	NR	Medium
Fritz et al. (2017) [39]	USA	CS	22 (m=50) Males:1, Females: 21	Fibromyalgia syndrome (FS)	SHRQ	SF-36	Humor was correlated with fewer physical symptoms in daily reports, but not with mental functioning (r=.35), and physical functioning (r=-0.9).	Humor was correlated with reduced psychological distress.	Medium
Helvik et al. (2006) [52]	Sweden	CS	343 (m=69) Age range: ≥20 (21-94) Males:188, Females:155	Hearing Impairment	SHQ-6	PGWB	The association between the PGWB index and sense of humor was significant (r=0.152, p< 0.01) and remained quite the same after the adjustment for the other variables.	NR	Good
Hyphantis et al. (2011) [49]	Greece	CS	176 Primary Sjögren’ s syndrome (SS) (n=40, m=55.8) Systematic lupus erythematosus, SLS (n=56, m=43.1), Healthy controls (n=80, m=56.2)	Primary Sjögren’s syndrome (SS)	DSQ	WHOQOL-BREF	Less use of humor defense (r=0.033, p<0.001) was significantly associated with impaired Physical HRQOL, independently of psychological distress in primary SS patients. There was not found significant association between humor and mental (r=0.162), social relations (r=0.287), and environmental HRQOL (r=0.109).	NR	Good
Lebowitz et al. (2011) [40]	USA	CS	46 (m=66.9) Males:19 Females:27	Chronic obstructive pulmonary disease (COPD)	CHS, SHRQ	SF-36	(1) The CHS was significantly correlated with mental health aspects of quality of life (r=.57, P < .001 but not with physical health aspects p the shrq was significantly correlated mental of quality life qol	(1) The CHS was inversely correlated with depression (r = -.47, P < .001) and anxiety (r=-.51, P < .001). (2) A moderate but nonsignificant relationship was evident between the CHS and number of days with an infectious illness (r= .34, P =.075). (3) The SHRQ exhibited a similar pattern of results, although the magnitude of correlations was smaller.	Medium
Lockwood & Yoshimura (2014) [41]	USA	CS	92 (m=58) Age range:20-91 Males: 40 Females: 52	Cardiovascular Disease	UHI, HO	WHOQOL-BREF, (Self-Integration Scale)	(1) Significant multivariate effects for the effects of humor types on psychological health (R = .40, F (3, 88) = 5.61, p < .05, R2 = .16) and social health (R = .36, F (3, 88) = 4.24, p < .01, R2 = .13). (2) Antidote humor positively related to increased reports of psychological (β = .26, p < .05) and social health (β = .34, p < .05). (3) Distancing humor negatively associated with psychological (β = −.35, p < .01) and social health (β=−.30, p < .05). (4) Conversation regulation humor also negatively related to psychological health (β = −.28, p < .05). (5) Humor orientation was associated with social health (β=.29, p < .01 and psychological health but was not associated with physical	(1) Conversation regulation humor (r=-.32, p < .05 and distancing humor p were negatively associated with relationship satisfaction.	Good
Merz et al. (2009) [42]	USA	CO	93 (Time 1, m= 49.2) - 74 (Time 2, m= 51.19)	Systemic sclerosis	CHS	HAQ	(1) Humor coping (HC) was significantly inversely associated with all outcomes cross-sectionally (disease severity, r=-0.171, p<0.05; pain, r=-0297, p<0.01; disability, r=-0.288, p<0.01; distress, r=-240, p< 0.05). Longitudinally, the only significant inverse relationship was between HC and pain (r=-0.238, p<0.05). (2) After controlling for covariates in cross-sectional hierarchical regression analysis, HC was a significant inverse predictor of disability; it was not a significant predictor of disease severity, pain, or distress.	NR	Good
Sousa et al. (2019) [51]	Portugal	CS	183 (m=59.17) Age range: >18	Chronic Kidney Disease (CKD)	MSHS	SWLG	(1) Satisfaction with life in general/personal wellbeing index was positively correlated with humor production and social use of humor (ρ=0.353, p<0.001); adaptive humor and appreciation humor (ρ=0.270, p<0.001) and attitude towards humor (ρ=0.211, p<0.001). (2) Humor production and social use of humor, and attitude towards humor had a positive effect on subjective happiness (β=0.239, p<0.05; β=0.165, p<0.001).	(1) Subjective happiness was positively correlated with humor production and social use of humor (ρ=0.476, p<0.001); adaptive humor and appreciation humor (ρ=0.387, p<0.001); and attitude towards humor (ρ=0.364, p<0.001). (2) Humor production and social use of humor, and attitude towards humor had a positive effect on subjective happiness (β=0.239, p<0.05; β=0.165, p<0.001). (3) Depression was negatively correlated with humor production, with social use of humor (ρ=-0.164, p<0.05) and with attitude towards humor (ρ=-0.240, p<0.01).	Good
Okajima et al. (2013) [30]	Japan	CR	83 (m=42.3) Males: 23 Females: 60	Primary lympedema	Brief COPE	SF-36, EQ-5D	Humor coping was associated with the mental aspect of HRQoL (SF-36, MCS) (β=1.89, p=0.005).	NR	Good
Owolabi (2010) [50]	Nigeria	CR	100 (m=58.9) Males: 43 Females: 57	Stroke	Laughter frequency Likert scale	SF-36, HRQOLISP	Laughter frequency affected psychological, cognitive and ecosocial domains of HRQOLISP in addition to physical functioning, vitality, mental health, social functioning, bodily pain and general health SF-36 subscales (0.000	NR	Good
Peter et al. (2014) [53]	Switzerland	CR	516 (m=53.1) Males: 372 Females: 144	Spinal cord injury	Brief COPE	WHOQoL BREF	Life satisfaction was strongly associated with humor coping (r = .30, p< .01).	Humor was significantly associated with self-efficacy (r=0.42, p < .01 and purpose in life p	Medium
Rinaldis et al. (2009) [48]	Australia	CO	1800 (m=65.07)	Colorectal cancer (CRC)	CCRC	FACT-C (Symptom checklist, Affect Balance Scale)	Humor, as coping strategy was not significantly associated with cancer-related QOL (r=0.00). Humor was significantly associated with positive affect (r=0.07, p<0.01), but not with psychological distress (r=-0.04).	NR	Good
Miller Smedema et al. (2010) [43]	USA	CR	242 (m=44.6) Age range: 18-81	Spinal cord injury	SHQ-6	SWBI, SLS	Sense of humor was associated with subjective well-being variables: life satisfaction (r=.240, p< .01) and quality of life (r=.078).	Sense of Humor was significantly associated with positive self-worth variables (Self-esteem, r=.382, p < .01 acceptance of disability r=.444)	Good
Svebak et al. (2006) [31]	Norway	CO	41 Non-survivors (n=17, m=68) Survivors (n=24, m=52.)	End Stage Renal Failure	SHQ-6	SF-12	Humor and GoL were significantly correlated (r=.37, p < .05 after controlling for age gender socioeconomic status and disease duration.)	NR	Good

Of the 18 studies that met the inclusion criteria, 13 had cross-sectional design, and five were longitudinal cohort studies. The included studies considered 10 categories of chronic diseases (Table 1): 1) cardiovascular diseases (e.g., coronary artery disease, CAD), 2) cancer (e.g., head and neck cancer), 3) chronic kidney disease (CKD), 4) chronic obstructive pulmonary disease (COPD), 5) hearing impairment, 6) primary Sjogren’s syndrome (SS), 7) spinal cord injury, 8) stroke, 9) systemic sclerosis, and 10) fibromyalgia syndrome (FS).

Methodological Quality of the Included Studies

The quality of studies ranged from five to nine stars. Among 18 studies, 14 studies showed good quality and four studies had medium quality. The quality appraisal of the included studies is shown in Tables 2, 3. 

**Table 2:** Newcastle-Ottawa quality assessment scale with modifications for cross-sectional studies Good quality: 7 - 10 stars Medium quality: 5 - 6 stars Poor quality: 0 – 4 stars

Authors (year)	Selection					Comparability		Outcome				
	Representative sample	Sample size	Non-responsese rate (>70)	Ascertainment of the exposure (risk factor)	Total (maximum score (=5)	Based on design and analysis	Total (maximum score =2)	Assessment of outcome	Statistical test	Total (maximum score =3)	Overall score (maximum score=9)	Quality*
Aarstad et al. (2011) [46]	*	*	*	**	5	**	2	*	*	2	9	Good
Beisland et al. (2013) [47]	*	*	*	**	5	**	2	*	*	1	9	Good
Forrette (2019) [38]	*			**	3	*	1	*	*	2	6	Medium
Fritz et al. (2017) [39]				**	2	*	1	*	*	2	5	Medium
Helvik et al. (2006) [52]	*	*	*	*	4	**	2	*	*	2	8	Good
Hyphantis et al. (2011) [49]	*			**	3	**	2	*	*	2	7	Good
Lebowitz et al. (2011) [40]	*			**	3	*	1	*	*	2	6	Medium
Lockwood & Yoshimura (2014) [41]	*		*	**	4	*	1	*	*	2	7	Good
Sousa et al. (2019) [51]	*	*	*	**	5	**	2	*	*	2	9	Good
Okajima et al. (2013) [30]	*	*	*	**	5	**	2	*	*	2	9	Good
Owolabi (2010) [50]	*	*			2	*	1	*	*	2	5	Medium
Peter et al. (2014) [53]	*	*		**	4	**	2	*	*	2	8	Good
Miller Smedema et al. (2010) [43]	*	*		**	4	**	2	*	*	2	8	Good

**Table 3:** Newcastle-Ottawa quality assessment scale for cohort studies Good quality: 7 – 9 stars Medium quality: 5 - 6 stars Poor quality: 0 – 4 stars

Authors (year)	Selection					Comparability		Outcome					
	Representativeness of the exposed cohort	Selection of the non-exposed cohort	Ascertainment of exposure	Demonstration that outcome of interest was not present at start of study	Total (maximum score (=4)	Comparability of cohorts on the basis of the design or analysis	Total (maximum score =2)	Assessment of outcome	Was follow-up long enough for outcomes to occur	Adequacy of follow-up of cohorts	Total (maximum score =3)	Overall score (maximum score=9)	Quality*
Aarstad et al. (2008) [45]	*	*	*		3	*	1	*	*	*	3	7	Good
Aarstad et al. (2005) [44]	*	*	*		3	**	2	*	*	*	3	8	Good
Merz et al. (2009) [42]	*	*			2	**	2	*	*	*	3	7	Good
Rinaldis et al. (2009) [48]	*	*		*	2	**	2	*		*	2	7	Good
Svebak et al. (2006) [31]	*	*			2	**	2	*	*	*	3	7	Good

Measurements of Humor

Humor was measured by a variety of self-reported instruments. The different components of humor reported in the included studies are presented in Table 4, while a brief description of each humor tool along with the references of relevant studies is presented in Table 5. Twelve tools were used to assess sense of humor, humor as a coping strategy, types of humor (e.g., self-enhancing), and humor-response behaviors (i.e., laughter frequency). The Svebak Humor Questionnaire (SHQ, SHQ-6) [54,55] was used in five studies and was the most used questionnaire assessing sense of humor. Humor as a coping strategy was measured by four tools, with Cope Inventory [56] being the most observed coping instrument. Additionally, a five-item Likert Scale was used by one study [50] to measure laughter frequency.

**Table 4:** Humor and quality of life (QoL) components in included studies

Study	Humor component				QoL component			
	Sense of humor	Coping	Types of Humor	Humor-response	General QoL	Physical	Mental/ psychological	Social/ environmental
Aarstad et al. (2011) [46]	√	√				√		
Aarstad et al. (2008) [45]		√				√		
Aarstad et al. (2005) [44]	√					√		
Beisland et al. (2013) [47]		√				√		
Forrette (2019) [38]			√			√	√	
Fritz et al. (2017) [39]	√					√	√	
Helvik et al. (2006) [52]	√						√	
Hyphantis et al. (2011) [49]		√				√	√	√
Lebowitz et al. (2011) [40]	√	√				√	√	
Lockwood & Yoshimura (2014) [41]	√		√			√	√	√
Merz et al. (2009) [42]		√				√	√	
Sousa et al. (2019) [51]	√						√	
Okajima et al. (2013) [30]		√					√	
Owolabi, (2010) [50]				√			√	√
Peter et al. (2014) [53]		√					√	
Rinaldis et al. (2009) [48]		√				√		
Miller Smedema et al. (2010) [43]	√						√	
Svebak et al. (2006) [31]	√				√			
Total Percentage (%)	9/18= 50%	9/18= 50%	2/18= 11%	1/18= 6%	1/18= 6%	11/18= 61 %	12/18= 67%	3/18= 17%

**Table 5:** Measurement tools used to examine humor

Humor instruments	Description	Humor component	Studies using this tool
Multidimensional Sense of Humor Scale (MSHS)	Consists of 24-item that comprises four factors: humor production, coping with humor, humor appreciation, and attitudes toward humor. Each item is rated on a 5-point Likert-type scale ranging from strongly disagree to strongly agree.	Sense of Humor	Sousa et al., 2019 [51]
Brief COPE Inventory	Consists of 28 items with a 4-Likert scale and comprises 14 subscales (self-distraction, active coping, denial, substance use, emotional support, instrumental support, behavioral disengagement, venting, positive reframing, planning, humor, acceptance, religion, and self-blame). Total scores per subscale range from 2 to 8.	Coping	Okajima et al., 2013 [30]; Peter et al., 2014 [53]
COPE Inventory	Consists of 13 scales assessing problem-focused coping, emotional-focused coping, avoidance-focused coping.	Coping	Aarstad et al., 2008 [45]; Aarstad et al., 2011 [46]; Beisland et al., 2013 [47]
Use of Humor Index (UHI)	Reflects five functions of humor: positive affect, negative affect, expressiveness, affiliation, and dominance. The items were measured on a 5-point Likert scale.	Types of Humor	Lockwood & Yoshimura, 2014 [41]
Humor Orientation Scale (HO)	Consists of 17 total Likert-Scale items designed to measure an individual’s predisposition to using humor regularly in social interaction. Each item is rated on a 5-point Likert-type scale ranging from strongly disagree to strongly agree.	Sense of Humor	Lockwood & Yoshimura, 2014 [41]
Coping Humor Scale (CHS)	Consists of seven items, each of which is a self-descriptive statement about the use of humor in coping with life stress. The items are rated in a 4-point Likert scale, with options ranging from 1 (strongly disagree) to 4 (strongly agree).	Coping	Lebowitz et al., 2011 [40]; Merz et al., 2009 [42]
Situational Humor Response Questionnaire (SHRQ)	Consists of 21 items, 18 situational items, and 3 generalized self-report items. SHRQ assesses the frequency of various mirthful behaviors (e.g., frequency of smiles, laughter).	Behavior	Fritz et al., 2017 [39]; Lebowitz et al., 2011 [40]
Defense Style Questionnaire (DSQ)	Estimates 25 ego defense mechanisms and consists of 8-item on a 9-point Likert type.	Defensive mechanism	Hyphantis et al., 2011 [49]
Svebak Humor Questionnaire (SHQ; SHQ-6)	Measures the cognitive, social, and affective dimensions of humor through three subscales: Metamessage sensitivity scale (SHQ-MS; 7 items), Personal liking of humor scale (SHQ-LH; 7 items), and Emotional expressiveness scale (SHQ-EE; 7 items). Each item is rated on a 4-point Likert scale. SHQ-6 is as= shorten version of the SHQ. Consists of 32 items rated on a -point Likert scale.	Sense of Humor	Aarstad et al., 2011 [46]; Aarstad et al., 2005 [44]; Helvik et al., 2006 [52]; Miller Smedema et al., 2010 [43]; Svebak et al., 2006 [31]
Humor Style Questionnaire (HSQ)	Assesses four styles of humor: two potential benign (self-enhancing, affiliative) and potential harmful uses of humor (aggressive, self-defeating)	Types of Humor	Forrette, 2019 [38]
Coping with Colorectal Cancer (CCRC)	Consists of 47 items and asks from participants to indicate how often they used each coping strategy in the past month.	Coping	Rinaldis et al., 2009 [48]
Laughter frequency	A 5 item Likert Scale	humor-response behavior	Owolabi, 2010 [50]

Measurements of Quality of Life

Many self-reported instruments were used to measure QoL. The SF-36 [34] was used in five studies and was the most used QoL instrument. A shortened form of SF-36, the Short Form 12 Item Health Survey (SF-12) [57], was also used by one study [31]. Additionally, five disease-related tools were used to assess the quality of life in specific chronic diseases, such as head and neck cancer (European Organization for Research and Treatment of Cancer Quality of Life Questionnaire Head and Neck Module; EORTC QLQ-H&N35) [58]. Ten additional QoL instruments were observed in the included studies. Different aspects of QoL measured in the included studies are presented in Table 4. A brief description of tools is presented in Table 6. Additionally, some researchers used supplementary tools to assess aspects of QoL, such as the Self-Integration Scale [59], the Depression, Anxiety and Stress Scale 21 (DASS - 21) [60], the Beck Depression Inventory [61], and the Psychosocial Adjustment to Illness Scale-Self Report (PAIS-SR) [62].

**Table 6:** Measurement tools used to examine quality of life (QoL)

GoL instruments	Description	QoL component	Studies using this tool
European Organisation for Research and Treatment of Cancer Quality of Life Questionnaire (EORTC QLQ-C30)	Assesses cancer patients' physical, psychological and social wellbeing. EORTC QLQ-C30 consists of 30 questions, resulting of multi-item scales and single items. Questions are answered in a four-point Likert format, except for questions about general health and general QoL (seven-point Likert format).	Disease related QoL	Aarstad et al. (2008) [45]; Aarstad et al. (2011) [46]; Aarstad et al. (2005) [44]; Beisland et al. (2013) [47]
European Organisation for Research and Treatment of Cancer Quality of Life Questionnaire Head and Neck Module (EORTC QLQ-H&N35)	Assesses the physical QoL patients with head and neck cancer. Contains multi-item scales and single items scales. Scale scores are transformed to a scale from 0 to 100 according to the EORTC scoring algorithm.	Disease related QoL	Aarstad et al.( 2005) [44]; Aarstad et al. (2008) [45]; Aarstad et al. (2011) [46]
Psychological General Well-Being Index (PGWB)	Consists of 22 items, rated on a 6-point scale, which assesses six dimensions: anxiety, depressed mood, positive well-being, self-control, general health, and vitality. Each dimension is summed and a total score (maximum=110) is obtained.	Health related QoL	Helvik et al., 2006 [52]
World Health Organization Quality-of-Life Scale - Brief (WHOQOL-BREF)	Generic QoL questionnaire comprising 26 questions and composed of four subscales (physical, psychological, social relationships, and environment). A total score or subscales score can be computed.	General QoL (or subscales' scores)	Hyphantis et al. (2011) [49]; Lockwood & Yoshimura (2014) [41]; Peter et al. (2014) [53]
The Short Form 36 Item Health Survey (SF-36)	Measures physical and mental health. Comprised of eight scales and provides two summary scores (physical - PCS score, and mental - MCS score).	Health related QoL	Okajima et al. (2013) [30]; Forrette (2019) [38]; Fritz et al. (2017) [39]; Lebowitz et al. (2011) [40]; Owolabi (2010) [50]
The Short Form 12 Item Health Survey (SF-12)	Is a shortened version of SF-36. Consists of 12 questions measuring eight physical and mental components.	Health related QoL	Svebak et al. (2006) [31]
Health Assessment Questionnaire (HAQ)	Assesses physical functioning in clinical populations and has been modified for patients with scleroderma. Contains a pain visual analog scale (HAQ-PVAS), and a disability index (HAQ-DI).	Disease related QoL	Merz et al. (2009) [42]
Satisfaction with life in general (SWLG)	Measures life satisfaction, an indicator of QoL. Consists of five items, rated on a 7-point Likert scale.	Life Satisfaction	Sousa et al. (2019) [51]
EuroQol-5 Dimension Questionnaire (EQ-5D)	Health-related questionnaire measuring five components: mobility, self-care, usual activities, pain/discomfort, and anxiety/depression. Answers are measured on a 5-point Likert scale.	Health related QoL	Okajima et al. (2013) [30]
Health-related quality of life (HRQOLISP)	HRQOLISP is a stroke-specific HRQOL questionnaire. Consists of two dimensions (physical and mental) and seven domains.	Disease related QoL	Owolabi (2010) [50]
Functional Assessment of Cancer Therapy – Colorectal (FACT-C)	FACT-C is part of the FACIT Measurement System, which assesses the health-related QoL of cancer patients and patients with other chronic diseases. A five point scale indicates the physical, social/family, emotional, functional, and colorectal-specific wellbeing.	Disease related QoL	Rinaldis et al. (2009) [48]
Sense of Well-Being Inventory (SWBI)	SWBI is a quay of life measure for people with disabilities. Consists of 36 items using a 4-point Likert scale.	Health related QoL	Miller Smedema et al. (2010) [43]
Satisfaction with Life Scale (SLS)	Measures the global life satisfaction and consists of five items rated on a 7-point Likert scale.	Life Satisfaction	Miller Smedema et al. (2010) [43]

Humor and physical QoL

**Figure 2:**
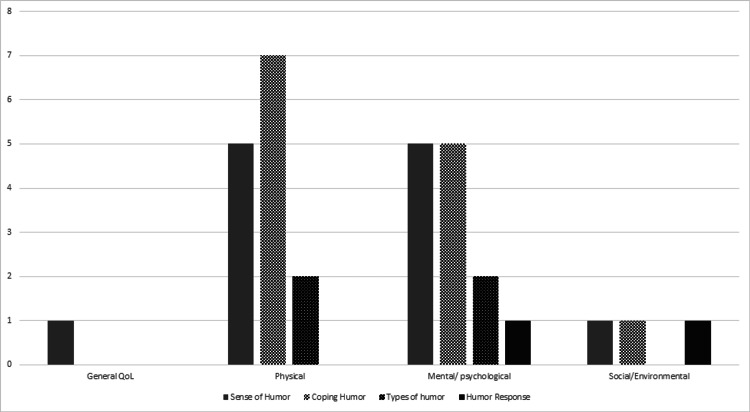
Number of studies with different humor components and quality of life (QoL) components

Across the 18 studies included in the review, 11 (61%) explored the relationship between different aspects of humor and physical QoL in patients with chronic diseases (See Table 1, Table 4, Figure 2).

Sense of Humor and Physical QoL

The relationship between sense of humor and physical QoL was investigated by five studies [39-41,44,46]. Two studies focused on head and neck cancer patients, and disease-related instruments were used [44,46]. The L scale (i.e., habitual tendency to enjoy or dislike comical situations) of SHQ [46] and the L and M scale (i.e., habitual sensitivity to humorous messages) of SHQ [44] were used. Findings showed that sense of humor was significantly associated with QoL subscales and the H&N&QOL sum score [46]. In Aarstad et al. [44] the total humor score predicted a higher QoL level after the adjustment for neuroticism (r=0.46; p<0.05). No significant associations were found between humor and physical QoL in three studies [39-41]. Moreover, sense of humor was significantly associated with social QoL [41] and general QoL [31].

Humor Coping and Physical QoL

Humor coping and physical QoL were examined in seven of the included studies [40,42,45-49]. Three studies investigated the relationship between coping humor and physical QoL using the EORTC QLQ-C30. Findings showed that coping humor correlated inversely with functional subscale in all three studies [45-47] and associated positively with symptom subscale in two studies [45,47]. The EORTC QLQ-H&N35, was significantly associated with coping humor [45,46]. In Hyphantis et al. [49] humor as a defense mechanism was significantly associated with physical QoL in patients with primary Sjögren’s syndrome. Coping humor and physical QoL were positively associated, but not significantly, in two studies involving COPD [40] and colorectal cancer patients [48]. Moreover, in a cohort study of systemic sclerosis patients [42] humor coping was inversely associated with physical QoL outcomes (i.e., disease severity, disability); however, after the application of controls, the only significantly remaining inverse relationship was between coping humor and disability. 

Other Measurements of Humor and Physical QoL

Forrette [38] examined humor styles (i.e., self-enhancing, affiliative, aggressive, and self-defeating) as predictors for physical QoL in patients with CAD. Results showed that as a group, humor styles were predictors of physical QoL. However, individual humor styles failed to significantly contribute predictive ability concerning physical QoL.

Humor and mental/psychological QoL

Across the 18 studies included in the review, 12 (67 %) explored the relationship between humor and mental QoL in patients with chronic diseases (Table 1, Table 4).

Sense of Humor and Mental/Psychological QoL

Six studies showed that sense of humor was positively related to mental/psychological components of QoL [39-41,43,51,52]. More specifically, in three studies, sense of humor was correlated with general psychological well-being in patients with hearing impairment [52], COPD [40] and cardiovascular diseases [41]. Fritz et al. [39] found no correlation between humor and mental functioning. The remaining two studies found significant correlation between the sense of humor and life satisfaction in patients with spinal cord injury [43] and CKD [51].

Coping Humor and Mental/Psychological QoL

Five studies examined coping humor and mental QoL and its components [30,40,42,49,53]. Positive associations were found between humor measurements and mental scale of SF-36 [30,40], distress [42], and life satisfaction [53]. No significant association was found between humor as defense mechanism and mental scale, social relations, and environmental HRQOL in the study of Hyphantis et al. [49].

Other Measurements of Humor and Mental/Psychological QoL

Two studies (11%) investigated how the specific types of humor related to QoL in patients with cardiovascular diseases [41,50]. In Lockwood and Yoshimura's study [41], findings indicated significant effects for the effects of humor types on psychological and social health. Specifically, antidote, distancing, and conversation regulation humor were examined. Findings showed that antidote humor was significantly correlated with both psychological and social QoL. Also, in the study of Forrette (2019) [38] humor styles were predictors of mental QoL in patients with CAD. In addition, one study (6%) examined the relationship between humor-response behavior and QoL, and particularly the effect of laughter frequency in post-stroke QoL [50]. Results showed that laughter frequency affected the psychological, cognitive, and eco-social domains of QoL.

Secondary Outcomes

As shown in Table 1, eight studies demonstrated associations between humor and other measurements [39-41,43,44,46,51,53]. Depression and anxiety scales were used in three studies. In Lebowitz et al. [40] humor coping was inversely correlated with depression and anxiety, while in Sousa et al. [51] depression was negatively correlated with various dimensions of sense of humor (i.e., humor production and social use of humor, attitude toward humor). Aarstad et al. [44] showed that a total humor score measured at diagnosis in head and neck cancer patients predicted a lower depression score and a lower “psycho” depression sub-score at follow-up. Also, in Fritz et al. [39] humor was correlated with reduced psychological distress.

Additional psychological outcomes were presented in three studies [43,51,53]. Peter et al. [53] found that humor was significantly associated with self-efficacy and purpose in life, while according to Miller Smedema et al. [43] sense of humor was significantly associated with self-esteem and acceptance of disability. Moreover, various aspects of humor were correlated (e.g., adaptive and appreciation humor) and presented a positive effect (e.g., attitudes toward humor) on subjective happiness [51]. Finally, Lockwood and Yoshimura [41] examined the association between types of humor and relationship satisfaction in cardiovascular patients. Results showed that relationship satisfaction was negatively associated with conversation regulation humor and distancing humor.

Discussion

In this systematic review, authors attempted to investigate the relationship between humor and quality of life in adults with chronic physical diseases. Eighteen studies were examined, each shedding valuable light on this intricate connection. The collective findings extracted from these studies revealed a noteworthy pattern. It became evident that various facets of humor displayed correlations with different dimensions of QoL among individuals dealing with chronic health conditions. Notably, humor consistently exhibited a positive association with the psychological and mental facets of QoL. This suggests that individuals who incorporated humor into their coping mechanisms tended to report higher scores in these domains of their quality of life. However, the relationship between humor and physical QoL exhibited a more complex picture, characterized by mixed results. These mixed outcomes suggest that humor's impact on the physical aspects of QoL may be influenced by various factors and might not have a universally positive effect. To the best of our knowledge, the current study represents the pioneering effort to conduct a systematic review investigating these specific associations within this particular population.

Humor and Mental/Psychological QoL

Our findings supported a positive association between different aspects of humor and psychological aspects of QoL. From a detailed standpoint, sense of humor and psychological and mental aspects of health were positively correlated in patients with hearing impairment [52], COPD [40], cardiovascular diseases [41], spinal cord injury [43] and CKD [51], but not in fibromyalgia patients [39]. Humor coping was also significantly associated with better psychological health in patients with COPD [40] and spinal cord injury [53] as well as in patients with primary lymphedema [30] and systemic sclerosis [42]. Only in the study of Hyphantis et al. [49], humor as a defense mechanism was not significantly associated with mental, social, and environmental QoL. Furthermore, laughter frequency, as a humor-response behavior, positively affected the psychological, cognitive, and social aspects of QoL [50].

Additional measures in included studies showed that sense of humor and coping humor were inversely associated with depression and anxiety scores [40,51]. Humor positively correlated with self-efficacy and purpose in life in spinal cord injury patients [53], with self-esteem and acceptance of disability in patients with the same disease [43], and with decreased psychological distress in fibromyalgia patients [39]. Finally, humor predicted subjective happiness in CKD patients [51].

These findings are in line with studies in non-clinical populations, which have reported that different measures of humor were positively associated with psychological and mental health [63], as well as with many specific psychological factors such as self-esteem [64], optimism and positive affect [65,66], self-efficacy [67], and a more positive orientation toward life [68]. The inverse relationship between humor and depression has been referred to by many studies in chronic patients [65,67,69]. However, Celso et al. [70] investigated humor coping, health status, and life satisfaction among older adults and found that humor coping was associated with better life satisfaction, but only for healthy older adults.

Yet, adaptive styles of humor were associated with better psychological and social QoL [38,41]. This finding agreed with a recent meta-analysis of 37 studies [71], which showed that health-promoting humor styles, such as self-enhancing and affiliative styles, were associated with better mental health, while self-defeating humor (i.e. making fun of oneself for the amusement of others) was negatively correlated with mental health. Also, studies have shown that aggressive humor (i.e. a hostile type of humor including sarcasm, and criticism) was negatively linked with different aspects of health and well-being [22,72,73]. Despite the fact that different types of humor seem to affect the physical and mental QoL in a different way, only two of the included studies examined how they were related with different aspects of well-being. So, further research is required to examine how adaptive and maladaptive humor styles are related to various aspects of QoL.

Humor and Physical QoL

On the other hand, the studies that investigated humor and physical QoL presented quite mixed results. Two studies showed that sense of humor was inversely associated with physical QoL subscales and predicted lower physical QoL in head and neck cancer patients [44,46]; however, with adjustment for neuroticism, the sense of humor predicted a higher cancer-related QoL [44]. Also, no significant association between sense of humor and physical QoL was observed in patients with cardiovascular diseases [41] and COPD [40].

Regarding coping humor, seven studies examined the association with physical QoL. Findings from three studies showed that humor coping was inversely correlated with the functional health of cancer patients [44-47] while positive correlations with symptom subscale (e.g., fatigue, pain, and nausea/vomiting) and general cancer-related QoL were observed [45,46]. Coping humor was associated with a better physical QoL in patients with primary Sjögren’s syndrome [49] and patients with systemic sclerosis [42]. Positive, but not statistically significant, associations were found between humor coping and physical health in COPD [40] and colorectal cancer patients [48].

Our results aligned with previous reviews of literature [22,74], which also concluded that the evidence regarding the effects of humor on physical QoL was limited and inconsistent. Similar results were presented in many studies in healthy and clinical populations, as some researchers found no association [75,76] or an inverse correlation with health QoL [77], while others supported that humor correlated with significant health outcomes, such as enhanced immune function [24], and reduced mortality [78].

The conflicting results could be attributed to various reasons. A first consideration concerned the methodological differences and limitations of the studies (e.g., small sample sizes, cross-sectional and correlational study design; instruments with different psychometric properties). In addition, the diseases’ specific characteristics (e.g., disease severity, pain, functional disabilities) and individual differences in humor (i.e., each person considers what is funny in a different way) or health condition (e.g., patients in different stages of different diseases) could have affected the findings of studies. Moreover, mediating factors (such as personality traits) may have influenced the association between humor and QoL [73]. For example, in the study of Aarstad et al. [44], coping humor predicted a higher physical QoL, only after the adjustment for neuroticism. Finally, context and cultural differences may affect the results of studies, as different cultures define humor, health, and disease in different ways [79].

The above findings can contribute to the design and implementation of humor and laughter interventions [80]. These interventions are commonly used to enhance psychological well-being and overall health, particularly within healthcare settings [81]. Empirical studies have shown the various positive effects of humor and laughter therapies, such as reduced stress and increased happiness, in healthy individuals [82] and clinical populations [83]. The results of a recent systematic review in older adults also showed that laughter and humor interventions enhance the well-being of participants [84]. Yet, studies focused on patients with chronic health issues such as diabetes [85] and arthritis [86] demonstrated positive health outcomes.

Limitations of the review

The present results were considered in light of certain methodological limitations that restricted our ability to fully interpret them. The first challenge of our systematic review was the absence of clear definitions for humor and QoL in the included studies. As Karimi and Brazier [7] argued, there was a considerable debate about the content of the terms “health”, “QoL” and “HRQoL” in the relevant literature, as the terms often overlapped or were used indistinguishably. Similar difficulties were also in humor’s definition, as humor is a multifaceted notion that was conceptualized differently by each researcher [73]. Furthermore, most studies had a cross-sectional design, therefore, no cause-effect relationship between humor and quality of life could be established. Some additional methodological weaknesses were related to the lack of control groups and the use of self-reported, and often self-conducted measures. In addition, the search of studies was conducted in the English language, which may have affected the generalizability of findings and led to a risk of language bias. Finally, a limitation of this study may be that the initial design was focused on a specific age group (i.e., older adults). However, authors considered that the large number of studies identified from databases (12,296), the present of a wide age range of participants in these studies (as perceived during the first and second screening), and the extensive hand-searching of eligible studies, grey literature (e.g., dissertations), and electronic databases, overcame the above limitation.

Future directions

In order to improve the quality of life of patients with chronic diseases, it is crucial to continue investigating the complex relationship between humor and QoL. The use of both self-rereported and objective measures (e.g., peer reports), the implementation of longitudinal research, the investigation of the effects of moderator variables (e.g., personality traits, stage of disease), and the presentation of outcomes for different age groups (e.g., adolescents, older adults) are recommended. Moreover, designing and conducting humor and laughter interventions, as well as conducting relevant reviews and/or meta-analyses specifically focused on adults with chronic health issues, is of great significance to obtain more robust findings regarding the effect of these treatments on patients’ QoL. Finally, it would be beneficial to conduct cross-cultural studies to assess the impact of culture on the humor-QoL relationship. Such research would contribute to determining the validity and generalizability of these results across cultures.

## Conclusions

Overall, the findings of this systematic review supported the connection between humor and QoL. Studies showed that people who used humor had a higher psychological, mental, and social quality of life, albeit the relationship between humor and physical aspects of QoL presented mixed and unclear results. Continuing research would provide additional clarity about the connection between different aspects of humor and QoL. Besides, the research on humor and QoL in chronic disease is crucial for the development of more effective assessment methods and the design of intervention programs, which would enhance the well-being of chronic patients, and would be very useful for the healthcare systems.
